# Dedifferentiated endometrioid carcinoma of the uterus : report of four cases and review of literature

**DOI:** 10.1186/s12957-016-1093-0

**Published:** 2017-01-10

**Authors:** Jiheun Han, Eun Young Ki, Sung Eun Rha, SooYoung Hur, Ahwon Lee

**Affiliations:** 1 Department of Hospital Pathology, Seoul St. Mary’s Hospital, College of Medicine, The Catholic University of Korea, 222, Banpo-daero, Seocho-gu, Seoul, 06591 Republic of Korea; 2 Department of Obstetrics and Gynecology, Seoul St. Mary’s Hospital, College of Medicine, The Catholic University of Korea, Seoul, Republic of Korea; 3 Department of Radiology, Seoul St. Mary’s Hospital, College of Medicine, The Catholic University of Korea, Seoul, Republic of Korea

**Keywords:** Dedifferentiated endometrioid carcinoma, Undifferentiated carcinoma, Endometrioid carcinoma

## Abstract

**Background:**

Dedifferentiated endometrioid adenocarcinoma (DEAC) is rare and is known to be more aggressive than high-grade endometrioid carcinoma. Differentiating between the two is important to provide appropriate treatment for patients.

**Case presentation:**

This is a retrospective study including four cases of DEAC of the uterus, which was diagnosed and treated in our Obstetrics and Gynecology department between January 2013 and December 2015. Clinical, pathological, and immunohistochemical staining features are discussed. Each tumor was composed of undifferentiated carcinoma (UC) and low-grade endometrioid carcinoma with abrupt transition between them. Two patients showed recurrence or progression within one month postoperatively and died at the last follow-up. An immunohistochemical study showed PAX-8, ER, PR, and E-cadherin expression in UC component.

**Conclusions:**

DEAC should not be underdiagnosed as conventional endometrioid adenocarcinoma due to its fulminant clinical course. Therefore, UC, including DEAC, should be further categorized to provide intensive treatment to improve patient survival.

## Background

Endometrial carcinoma is the most common gynecologic malignancy in developed countries [1]. It comprises several pathological subtypes, such as endometrioid, mucinous, clear cell, mixed cell, undifferentiated, and dedifferentiated carcinoma[2]. The last two entities are recently defined to distinguish them from other less aggressive tumors, therefore, providing proper treatment for patients.

Undifferentiated carcinoma (UC) of the endometrium is defined as a malignant neoplasm with no differentiation. It displays solid patternless growth and has worse clinical outcome than high-grade endometrioid adenocarcinoma. It is often observed admixed with differentiated endometrioid carcinoma (grade 1/2), which is referred to as dedifferentiated endometrioid adenocarcinoma (DEAC). The biologic features of DEAC are known to be determined by UC component even when this component represents 20% of the entire neoplasms, thus, with aggressive outcome [3].

In the current International Federation of Obstetrics and Gynecology (FIGO) grading system of endometrioid adenocarcinoma, tumors are graded by the proportion of solid components within a tumor, without further details on the histologic features of solid areas, resulting in misdiagnosis of DEAC as FIGO grade 2 or 3 endometrioid carcinoma. However, differentiating between the two is important in providing appropriate treatment options for patients. The present study reports four cases diagnosed with DEAC and reviews the literatures updated on the clinical, radiological, and pathological DEAC characteristics of the uterus.

## Case presentation

This is a retrospective study including four cases of DEAC of the uterus, which was diagnosed and treated in our Obstetrics and Gynecology department between January 2013 and December 2015. During this period, 155 cases of endometrial carcinoma were initially diagnosed and of which four patients (2.6%) were diagnosed with DEAC in our hospital. Clinical, pathological, and immunohistochemical staining features are outlined below and in Table 1, Table 2, and Table 3.

**Table 1:** Clinical and pathologic features of four patients with dedifferentiated endometrioid adenocarcinoma (DEAC) of uterus

	Case 1	Case 2	Case 3	Case 4
Clinical features				
Age at diagnosis, years	77	54	60	52
Age at menopause, years	60	51	52	Not applicable
Presentation	Postmenopausal bleeding	Postmenopausal bleeding	Postmenopausal spotting	Perimenopausal bleeding
Initial diagnosis	Poorly differentiated carcinoma	Endometrioid adenocarcinoma, FIGO grade 3	Pap smear: adenocarcinoma vaginal biopsy: poorly differentiated carcinoma	Leiomyoma
Imaging findings	MRI: 8.6 × 3.7 cm sized heterogeneously enhanced mass on CET1WI with involvement of the cervical stroma	MRI: 5.0 × 3.6 cm sized lobulated mass filling endometrial cavity with slightly high SI in T2WI and poor enhancement than adjacent myometrium	MRI: 7.2 × 3.5 cm sized lobulated mass which showed high SI on T2WI and low SI on T1WI with heterogeneous enhancement	CT: 10 cm sized low density mass in the endometrial cavity and cervical canal
Surgical management	TH/BSO/PLND	TH/BSO/PLND/PALND	Wide cuff TH/BSO	TH/BSO/PLND/PALND
Operative findings	Cancer extension to cervix	Invasion to superficial myometrium	Cancer extension to cervix and vaginal wall extension	Necrotic mass filling endometrial cavity with protruding through cervical canal
Postoperative management	Refused	Not needed	Chemotherapy (CDDP + ADR + CTX) and EBRT(50 Gy) + ICR(20 Gy/4fx)	EBRT(50 Gy) + ICR(20 Gy/4fx)
First postoperative recurrence or progression	1 month	None	1 month	None
FIGO surgical stage	II	IA	IIIB	II
Clinical history	Hypertension	Hypertension	Hypertension	None
Family history	None	None	Gastric cancer (mother)	None
Status at last follow-up	DOD (7 weeks)	NED (19 months)	DOD (10 months)	NED (39 months)
Pathologic features				
Tumor location	Fundus, body, lower uterine segment, cervix	Body	Body, lower uterine segments	Body, lower uterine segment
Tumor grade	G2 (80%) + UC (20%)	G1 (70%) + UC (30%)	G1 (10%) + UC (90%)	G2 (40%) + UC (60%)
Myometrial invasion	Full thickness of myometrium	<1/2 of myometrium	<1/2 of myometrium	>1/2 of myometrium
Lymphovascular space invasion (LSI)	Present	Present	Present	Present
Cervical stromal invasion	Present	Absent	Present	Present
Ovaries and fallopian tubes	Unremarkable	Unremarkable	Unremarkable	Unremarkable

*CET1WI* contrast-enhanced T1-weighted image; *SI* signal intensity; *T2WI* T2-weighted image; *T1WI* T1-weighted image; *TH* total hysterectomy; *BSO* bilateral salpingo-oophorectomy; *PLND* pelvic lymph node dissection; *PALND* para-aortic lymph node dissection; *EBRT* external beam radiation therapy; *ICR* intracavitary radiation; *DOD* die of disease; *NED* no evidence of disease; *UC* undifferentiated carcinoma; *G1* FIGO grade 1 endometrioid adenocarcinoma; *G2* FIGO grade 2 endometrioid adenocarcinoma; *CDDP* cisplatin; *ADR* adriamycin; *PTX* paclitaxel; *CTX* cyclophosphamide

**Table 2:** Results of immunohistochemical stains of the four cases

IHC stain	Case 1		Case 2		Case 3		Case 4	
	UC	DC	UC	DC	UC	DC	UC	DC
CK 8/18	Diffuse, perinuclear dot-like	Diffuse, cytoplasmic	Diffuse, perinuclear dot-like, and cytoplasmic	Diffuse, cytoplasmic	Negative	Diffuse, cytoplasmic	Focal, perinuclear dot-like, and cytoplasmic	Diffuse, cytoplasmic
Pancytokeratin	Diffuse, perinuclear dot-like	Diffuse, cytoplasmic	Diffuse, perinuclear dot-like < cytoplasmic	Diffuse, cytoplasmic	Focal, perinuclear dot-like < cytoplasmic	Diffuse, cytoplasmic	Focal, perinuclear dot-like < cytoplasmic	Diffuse, cytoplasmic
EMA	Negative	Diffuse, cytoplasmic	Diffuse, perinuclear dot-like, and cytoplasmic	Diffuse, cytoplasmic	Focal, perinuclear dot-like, and cytoplasmic	Diffuse, cytoplasmic	Focal, perinuclear dot-like, and cytoplasmic	Diffuse, cytoplasmic
Vimentin	Focal	Negative	Diffuse	Diffuse	Diffuse	Diffuse	Diffuse	Focal
ER	Negative	Positive	Negative	Positive	Negative	Negative	Negative	Positive
PR	Negative	Positive	Negative	Positive	Negative	Negative	Negative	Positive
E-cadherin	Negative	Diffuse	Negative	Focal	Negative	Diffuse, weakly	Negative	Diffuse
PAX-8	Negative	Diffuse	Negative	Diffuse	Negative	Diffuse	Diffuse	Diffuse

Proportion of tumor cells are >50%, diffuse; 10 ~ 50%, focal; <10%, negative; *EMA* epithelial membrane antigen; *ER* estrogen receptor; *PR* progesterone receptor

**Table 3:** Cases of dedifferentiated endometrioid adenocarcinoma (DEAC) with clinical and pathologic features

Author	Number of cases	Age, years	Surgical operation (cases)	Stage (cases)	Component of tumor	Marker expression of UC component	Adjuvant treatment (cases)	Survival outcome (cases)
Silva, EG (2006) [3]	25	51 (median) (range: 30–82)	TH+ BSO (24)	I (14) II (1) III (6) IV (4)	Low grade (10–80%) + UC(20-90%)	Keratin (13 out of 15, focal or diffuse) EMA (all) Neuroendocrine marker (4 out of 15)	Chemotherapy (18) radiation (4)	DOD (15) (median: 7 months) AWPD (6) (6 ~ 8 months) NA(3)
Shen, Y (2012) [18]	1	51	TH + BSO + PLND	II	Low grade (80%) + UC(20%)	Negative for EMA focally positive for CK7, CK18	Vaginal radiation + chemotherapy (CDDP+ DTX + Taxanes)	NA
Vita, G (2011) [19]	1	45	TH + BSO	IIIA	Low grade (60%) + UC(40%)	Positive for cytokeratins and EMA	Chemotherapy (CDDP + ATC + Taxanes)	NA
Wu, ES (2013) [20]	1	62	NA	NA	NA	NA	Radiation + hormone therapy (Megace alternating with Tamoxifen)	AWPD (3 months)
Berretta, R (2013) [21]	1	67	NA	IV	NA	Positive for keratin and negative for neuronal markers	Chemotherapy (CBDCA + Taxol)	NA
Park, SY (2014) [22]	1	55	TH + USO + PLND	IB	Low grade (40%) + UC(60%)	Focally positive for CK and EMA	Chemotherapy (PTX + CDDP+DOXO)	DOD (7 months)
Li, Z (2016) [15]	13	61 (median)	NA	III/IV (12)	NA	Pancytokeratin (10 out of 13) Cam5.2 (8 out of 13) EMA (8 out of 13, weak and patchy) PAX-8 (1 out of 13)	Chemotherapy and radiation (13)	Recurrence or metastasis within 3 years of diagnosis (12) Disease-free after 3 years of diagnosis (1)
Soyama, H (2016) [23]	1	41	Supravaginal hysterectomy + USO+partial resection of ileum	IVB	NA	NA	Chemotherapy alone	DOD (7 months)
Rabban (2016) [24]	1	50	TH + BSO+PLND	IA	G1(60%) + UC(40%)	Negative for EMA, keratin, PAX-8	Untreated	Progression after 10 months of surgery

*TH* total hysterectomy; *BSO* bilateral salpingo-oophorectomy; *PLND* pelvic lymph node dissection; *DOD* die of disease; *AWPD* alive with progressive disease; *USO* unilateral salpingo-oophorectomy; *NA* not available; *CDDP* cisplatin; *DTX* docetaxel; *ATC* anthracycline; *CBDCA* Carboplatin; *PTX* paclitaxel; *DOXO* doxorubicin

### Case 1

**Fig. 1:**
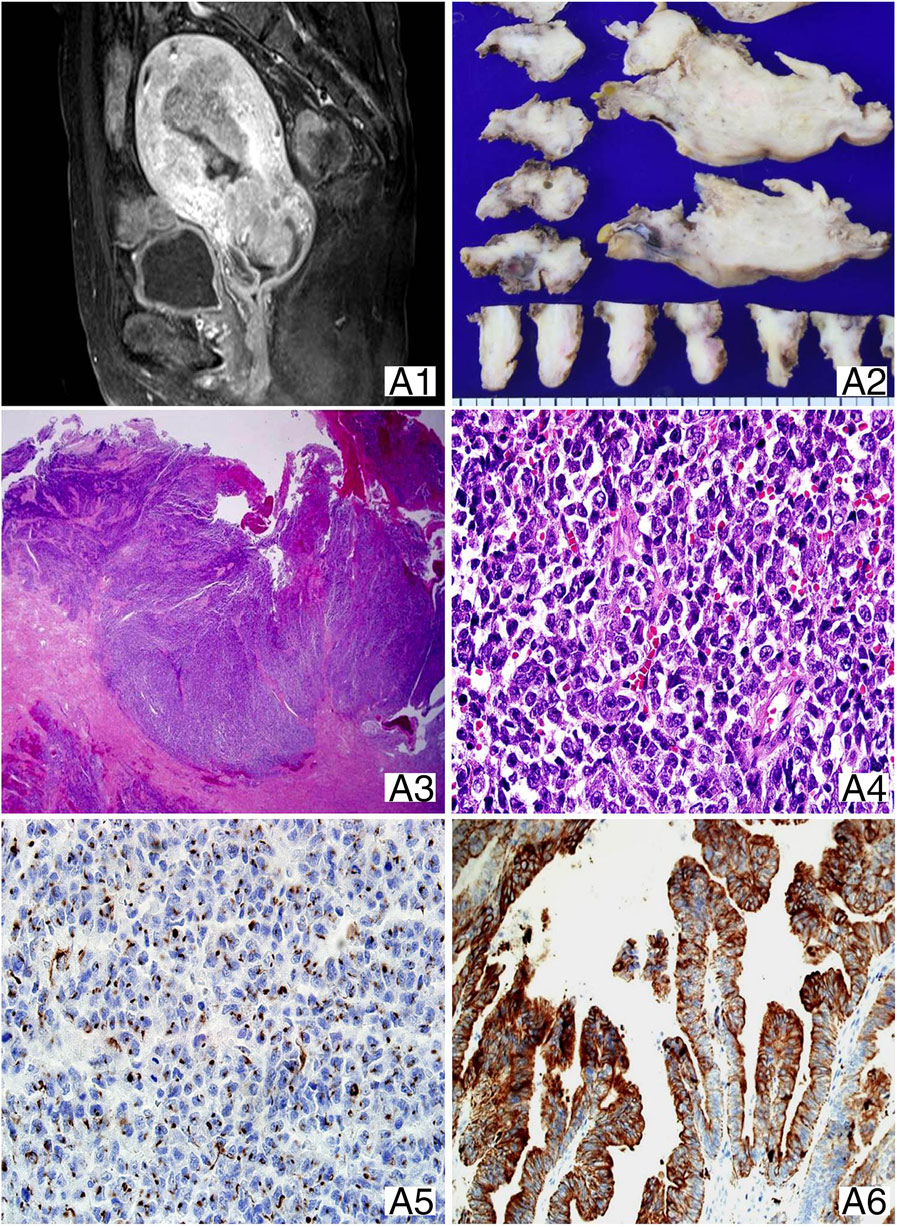
Case 1. (*A1*) Sagittal contrast-enhanced T1-weighted MRI shows heterogeneously enhanced mass filling the endometrial cavity with cervical stromal invasion. (*A2*) The tumor shows white infiltrative lesion with necrosis involving lower uterine segment and uterine body, extending to cervix. The UC component (*A3*, ×2.5) infiltrates the full thickness of the lower uterine segment and arranged in patternless with extensive necrosis and hemorrhage. The tumor (*A4*, ×400) comprises monotonous cells with moderate pleomorphism, prominent nucleoli, and high mitotic rates. Cytokeratin 8/18 (CK8/18) expressed as perinuclear dots in the UC component (*A5*, ×400) and showed diffuse cytoplasmic expression in differentiated component (*A6*, ×200)

A 77-year-old woman (body mass index [BMI], 19.6 kg/m^2^) presented with 1 week postmenopausal bleeding, which was preceded by vaginal spotting for 1 year and concomitant genital itching that had continued for 2 weeks. Transvaginal ultrasound revealed abnormally thickened endometrium (1.47 cm) and biopsy was recommended, but she refused. After 2 weeks, follow-up ultrasonography showed remarkably increased endometrium thickness (2.36 cm) with a 7 cm mixed-echoic lesion within the endometrial cavity. Endometrial curettage revealed poorly differentiated carcinoma. Magnetic resonance imaging (MRI) showed 8.6 × 3.7 cm heterogeneously enhanced mass on contrast-enhanced T1-weighted images (CET1WI) (Fig. 1a). In addition, tumor involvement of the cervical stroma was observed on CET1WI. The patient subsequently underwent total hysterectomy (TH) with bilateral salpingo-oophorectomy (BSO) and pelvic lymphadenectomy. The surgical specimen of the uterus showed white gray polypoid mass filling the endometrial cavity (Fig. 1b). Upon microscopic examination, the tumor with high cellularity and patternless growth without glandular differentiation invaded the full thickness of the lower uterine segment (Fig. 1c) extending to the cervical stroma. The tumor consisted of medium–sized monotonous cells with brisk mitosis (5 per high-power field (HPF)) and atypical mitotic figures (Fig. 1d). Moderately differentiated endometrioid adenocarcinoma component with squamous differentiation comprised the major proportion of the tumor mass of the uterine body and fundus, which invaded more than one half of the myometrial thickness. No lymph node (LN) metastasis was found. Cytokeratin 8/18 (CK8/18) and pancytokeratin (CK AE1/AE3) expressed both components but in different patterns. In the UC component, they were expressed as dot-like patterns, whereas they showed cytoplasmic expression in differentiated component (Figs. 1e, f). Epithelial membrane antigen (EMA), estrogen receptor (ER), progesterone receptor (PR), E-cadherin, and PAX-8 were expressed in the differentiated component alone (Table 2). Based on the FIGO system, the patient had stage II. She was recommended adjuvant external beam pelvic RT (EBRT), but she refused. A month after the surgery, she presented with difficulty in urination. Abdominal pelvic computed tomography (CT) revealed multiple, large, peritoneal seeding masses, and multiple lymph node metastases in the external and internal iliac chains with large amount of ascites which was positive for malignant cells on cytologic examination. She died 7 weeks later after surgery due to tumor lysis syndrome.

### Case 2

**Fig. 2:**
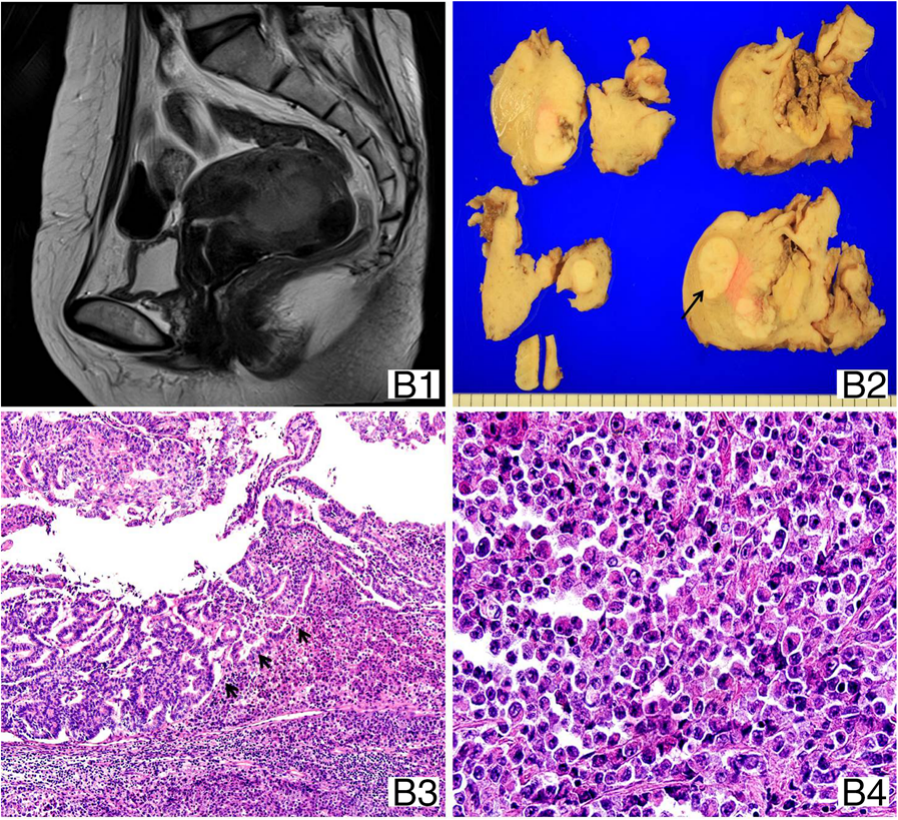
Case 2. (*B1*) Sagittal T2-weighted MRI shows a large polypoid endometrial mass with superficial infiltration of myometrium. (*B2*) Gross photograph shows a polypoid mass compacting the endometrial cavity. Well-circumscribed round mass, submural leiomyoma is seen (*arrow*). (*B3*, ×100) The tumor comprises moderately differentiated endometrioid adenocarcinoma and UC with abrupt transition (*arrows*) between them. (*B4*, ×400) The UC cell component shows discohesive rhabdoid feature

A 54-year-old postmenopausal woman (BMI: 22.5 kg/m^2^) was referred to our hospital after being diagnosed with endometrioid carcinoma FIGO grade 3 on biopsy at a local hospital. She experienced vaginal bleeding for 3 months. She had menopause at 51 year old hypertension history. MRI showed a 5.0 × 3.6 cm lobulated mass filling the endometrial cavity, which showed slightly high signal intensity (SI) on T2-weighted imaging (T2WI) and showed poor enhancement compared with the adjacent myometrium (Fig. 2a). Mildly enlarged pelvic LNs were noted in both external iliac areas. TH with BSO, pelvic lymphadenectomy, and para-aortic lymphadenectomy were performed. The mass was bulky and filled the entire endometrial cavity on gross examination (Fig. 2b). Microscopically, well-differentiated endometrioid adenocarcinoma and UC with abrupt transition were found between them (Fig. 2c). The UC component showed a solid monomorphic discohesive cell growth, showing no differentiation except for the presence of some rhabdoid cells (Fig. 2d). The invasion depth was less than one half of the myometrium, and LN metastasis was not observed. CK8/18, CK AE1/AE3, and EMA showed diffuse cytoplasmic expression in both components, and perinuclear cytokeratin dots were also observed in the UC component. The ER, PR, E-cadherin, and PAX-8 expressions were recognized in the differentiated component alone (Table 2). The final FIGO stage was IA, and adjuvant treatment was not performed. The patient has been disease-free for 19 months after the initial diagnosis.

### Case 3

**Fig. 3:**
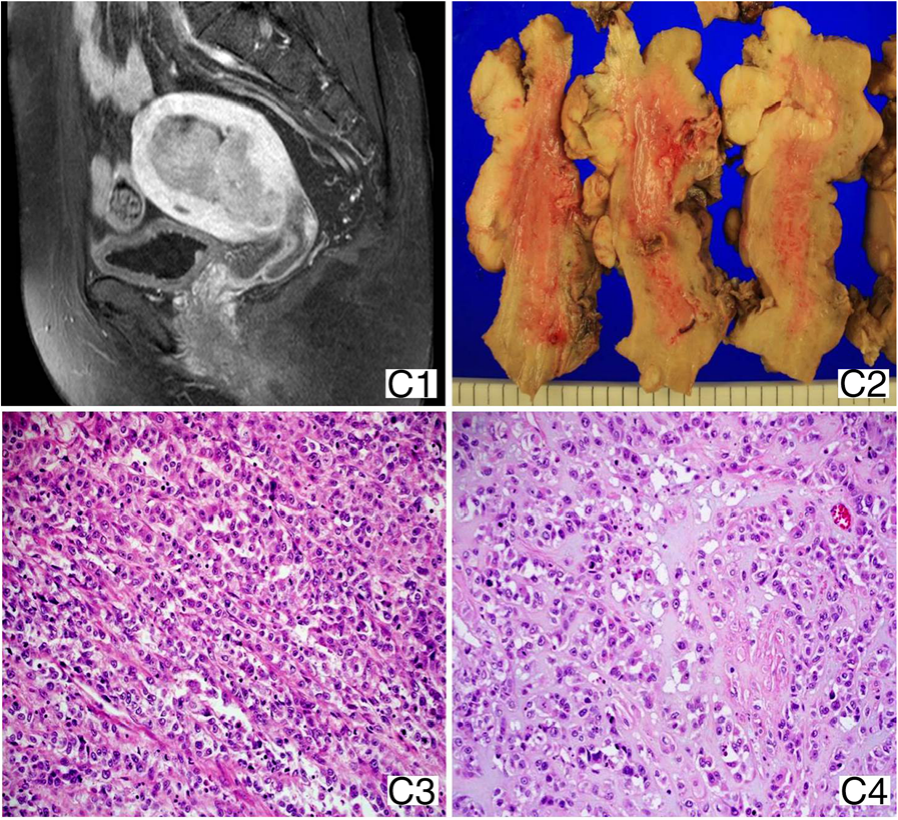
Case 3. (*C1*) Sagittal contrast-enhanced T1-weighted MRI shows a large polypoid mass lesion filling the endometrial cavity. (*C2*) The tan polypoid mass involving more than one half of the myometrium. (*C3*, ×200) A delicate fibrovascular septa separating discohesive cells into vague alveolar nests are found. (*C4*, ×200) Focal areas of UC component shows myxochondroid stroma with embedded tumor cells, resembling the cartilage

A 60-year-old postmenopausal woman (BMI: 25.3 kg/m^2^) with a chief complaint of vaginal spotting for 1 year visited a local gynecologic clinic. She had menopause at 52 years old. Colposcopy showed necrotic tissue with bleeding at the right vaginal wall, and she was referred to our hospital for further evaluation. Ultrasonography showed an echogenic mass in the endometrial cavity. The MRI revealed a 7.2 × 3.5 cm lobulated mass extending to the right above the endocervix (Fig 3a). The mass showed high SI on T2WI and low SI on T1WI with heterogeneous enhancement. Another 5 cm, elongated mass involving the lower vagina was also found. Vaginal biopsy revealed poorly differentiated carcinoma, and cytologic test of the endocervix revealed adenocarcinoma. The patient underwent wide cuff TH with BSO, and the separated additional vaginal mass was not removed. The surgical specimen showed a polypoid mass with necrosis and hemorrhage, which invaded more than one half of the myometrium of the uterus (Fig. 3b), extending to the cervix. Most of the tumor comprised discohesive cell growth with a solid sheet pattern on microscopic examination, suggesting a UC component. They were occasionally segregated by delicate fibrovascular septa forming a vague alveolar pattern (Fig. 3c). The UC component showed small amount of myxochondroid stroma, reminiscent of cartilage (Fig. 3d). A small proportion (10%) of tumor consisted of low-grade endometrioid adenocarcinoma juxtaposed with UC component. The histological finding of the UC component was similar to that of the lesion in the vaginal wall, suggesting vaginal metastasis (drop metastasis). ER and PR were not expressed in both UC and differentiated component on immunohistochemical staining (Table 2). One month after the surgery, postoperative positron emission tomography-CT revealed multiple metastatic lymphadenopathies in para-aortic, retrocaval, paracaval, both common and external iliac chains, and superficial inguinal areas with bone metastasis. The patients was treated with sequential chemoradiation therapy, which comprised of chemotherapy (cisplatin + Adriamycin + cyclophosphamide) followed by EBRT, with a total dose of 50 Gy was administered for 5 weeks to the whole pelvis, vagina, and both inguinal lymph nodes, and high-dose intracavitary radiotherapy (ICR) (20 Gy in fractions). She presented with dyspnea 2 months after radiation therapy, and clinical examination revealed lung metastasis. She died 3 weeks later (10 months after surgery).

### Case 4

**Fig. 4:**
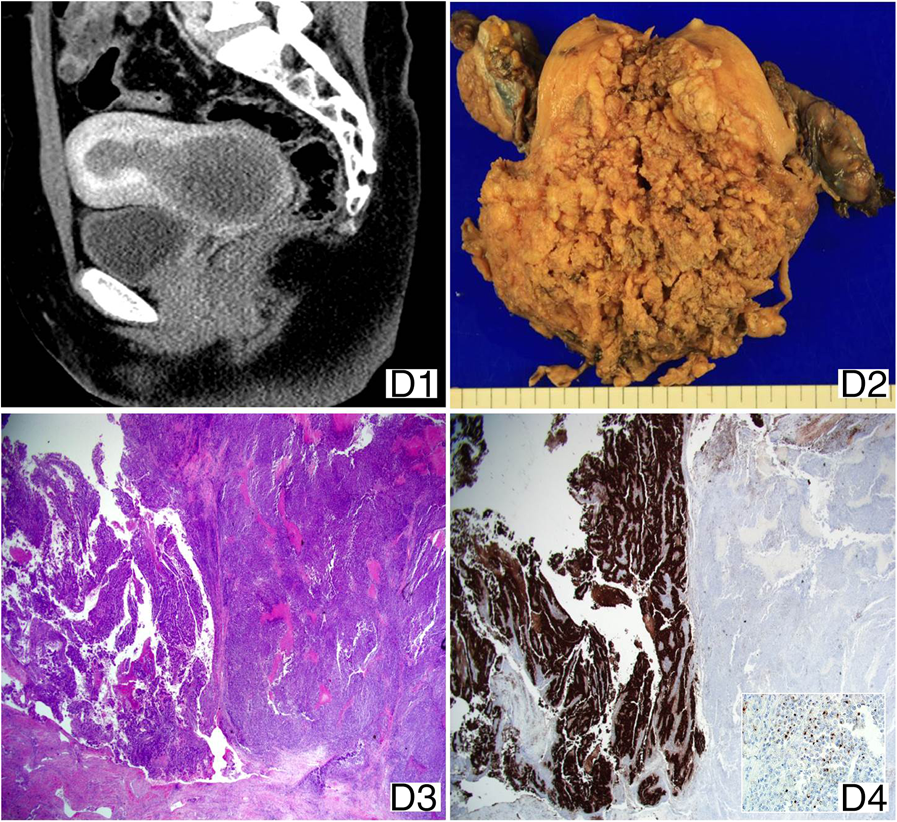
Case 4. (*D1*) Sagittal contrast-enhanced CT shows a bulky hypodense mass filling the endometrial cavity. (*D2*) The large outbulging mass with friable surface. (*D3*, ×2.5) A sharp border between differentiated and UC component and EMA (*D4*, ×2.5 and ×400) shows strong cytoplasmic expression in the former and focal dot and cytoplasmic expression in the latter

A 52-year-old nulligravid, perimenopausal woman (BMI 22.4 kg/m^2^) was admitted to our hospital for treatment of leiomyoma, manifested as 10 cm low-density mass in the endometrial cavity and cervical canal on pelvic CT at a local clinic (Fig. 4a). She experienced intermittent vaginal bleeding 3 years prior. During surgery, the surgeon observed a 10 cm necrotic mass protruding through the cervical canal, which seemed to fill the entire endometrial cavity. Frozen section revealed malignancy. Surgical stage was FIGO stage II, due to cervical stromal invasion. Any enlarged lymph node was not recognized. Hence, the patient was treated with bilateral salpingectomy with pelvic and para-aortic lymph node dissection. The fungating mass filling the entire endometrial cavity was recognized on gross examination (Fig. 4b). The tumor comprised of conventional endometrioid adenocarcinoma (FIGO grade 2) and UC with sharp border between them (Fig. 4c). CK8/18, CK AE1/AE3, and EMA were expressed focally as dot-like and cytoplasmic pattern in UC component (Fig. 4d). PAX-8 was diffusely expressed in both components (Table 2). The patient was treated with adjuvant EBRT in the whole pelvis with a total dose of 50 Gy in 28 fractions followed by vaginal cylinder ICR (20 Gy/4 fractions). No evidence of recurrence or progression of disease was found after 39 months after surgery.

## Discussion

Because DEAC has been recently recognized, the incidence rate is not established. The incidence of undifferentiated carcinoma is known to be 1 to 9% [4, 5]. In several retrospective studies, 37 to 87% of UC were admixed with endometrioid adenocarcinoma [3, 5, 6]. In our institution, 2.6% of patients (four out of 155 patients) with endometrial carcinoma were diagnosed with DEAC between 2013 and 2015.

A dualistic model, proposed by Bokhman [7] based on clinical and epidemiological observations, has been known to be correlated with endometrial carcinoma classification based on histopathological subtypes. Predominant form of type 1 endometrial carcinoma is low-grade endometrioid adenocarcinoma, whereas type II encompasses high-grade endometrioid adenocarcinoma, and most non-endometrioid histologic subtypes. However, increasing evidence supports that the binary classification is imperfect [8], and DEAC may be one of the representative entities. Type I tumors are known to be associated with unopposed estrogen stimulation, whereas type II tumors are commonly described as estrogen independent. The established risk factors for type I endometrial carcinomas are obesity, unopposed estrogen therapy use, nulliparity, early menarche, late menopause, oral contraceptive use, and smoking, whereas little is known regarding the risk factors for type II tumors. A recent study [9] showed that most classical endometrial cancer risk factors were also associated with type II tumors, suggesting that the etiology of type II tumors may not be completely estrogen independent. In the same context, three of four patients of our cases were postmenopausal, older (≥54 year old) women, and the other perimenopausal woman was nulliparous. Three patients of our cases had hypertension as underlying disease. Giordano et al. [10] showed that the majority of patients with malignant endometrial polyps had risk factors, such as hypertension, obesity, and unopposed estrogen therapy, for the development of endometrial carcinoma.

In all our cases, DEAC could not be diagnosed on biopsy or curettage and imaging studies, but could only be correctly diagnosed by pathological examination of the surgical specimen. Two cases were initially diagnosed as poorly differentiated carcinoma, and one case was diagnosed as FIGO grade 3 endometrioid adenocarcinoma by endometrial biopsy or curettage. A recent study [11] showed that preoperative endometrial biopsy or curettage is sensitive overall for detecting endometrial cancer, but sensitivity decreases with high-risk histology endometrial cancer. In addition, interobserver reproducibility in diagnosing high-grade endometrial carcinomas has been known to be worse than that of low-grade tumors [12]. Therefore, if preoperative examination suggests high-grade endometrial carcinomas, the surgeon should proceed to a more thorough surgical staging.

To correctly diagnose DEAC, recognizing the UC component is important. Recent studies [5, 13] emphasized that there is reproducible, distinctive histological features of UC, which are confirmed in all present cases. Although histological findings of UC can overlap with that of high-grade endometrioid adenocarcinoma, differences were found between them. In UC, tumor cells lack intercellular cohesion and arranged in patternless solid sheets without gland formation, which were occasionally admixed with rhabdoid cells. In contrast, high-grade endometrioid adenocarcinoma has at least a foci of gland formation of cohesive cells with rare or no rhabdoid cells. In addition, the tumor cells of UC in our cases also tended to have larger nuclei with more prominent nucleoli than conventional endometrioid adenocarcinoma. When UC are juxtaposed with low-grade endometrioid adenocarcinoma, such as in DEAC, a sharp boundary is noted between them, whereas a seamless transition from glandular component to solid area is observed in high-grade endometrioid adenocarcinoma. Immunochemical studies are also helpful in making a differential diagnosis. Among the epithelial markers, EMA and CK18 are known to be strongly and diffusely stained in the solid area of high-grade endometrioid carcinoma, but are focal or weak in the UC component [6]. In the previous case reports, expression of EMA in the UC components showed relatively inconsistency (Table 3), and our cases also showed variable expression of EMA and CK18 (Table 2). However, we found that expression of EMA and CK18 were perinuclear dot-like pattern in UC component, whereas diffuse cytoplasmic pattern in differentiated component in all cases. Ramalingam et al. [14] suggested PAX-8 to be the most effective immunomarker to distinguish UC from the solid component of either endometrioid carcinoma or serous carcinoma. In the present study, three of 4 cases expressed PAX-8 in the differentiated component alone, whereas the other one also expressed PAX-8 in the UC component. ER, PR, and E-cadherin are known to be retained in conventional endometrioid adenocarcinoma [6, 15, 16], but not in the UC, which were consistent with our cases. Therefore, perinuclear dot-like staining of cytokeratin and EMA, loss of expression of PAX-8, ER, PR, and E-cadherin would be helpful to distinguish UC from conventional endometrioid adenocarcinoma.

Differentiating between them is important because DEACs have fulminant clinical outcomes and poorer prognosis than high-grade endometrioid carcinoma. Several reports of DEAC cases with clinical feature, are summarized in Table 3.

In our cases, the proportion of UC component ranged from 20 to 90% (Table 1), which did not seem to be associated with clinical outcomes. In addition, the involvement to the lower uterine segment and cervix occurred in the UC component in all cases.

The morphologic appearance of DEACs suggest very broad differential diagnoses, including not only high-grade endometrioid adenocarcinoma, but also unclassified sarcomas, malignant mixed Müllerian tumors (MMMT), and rhabdoid tumor.

The UC component in DEAC may be negative or focal positive for keratin in immunohistochemical staining, and it can be misdiagnosed as sarcoma. Most sarcomas in the uterus comprise spindle cells with muscular differentiation and seldom comprise epithelioid cells only. Immunostaining with markers for muscular differentiation, such as desmin, caldesmon, and SMA may help to distinguish between them because DEACs are negative for these markers.

MMMT is a biphasic tumor that usually contains carcinoma and sarcomatous components. However, both components are high-grade in MMMT, whereas the gland-forming component is low-grade (grade 1/2), and no mesenchymal component is found in DEACs. Case 3 had focal areas with myxoid stroma mimicking heterologous component of MMMT. MMMTs typically occur in older women, whereas DEACs may occur in young patients. Moreover, these two entities may have distinct biology; MMMT is usually not associated with microsatellite instability, however, a proportion of DEACs appear to be associated with microsatellite instability [17].

Our cases had various proportions of rhabdoid cells, and if these are prominent, DEACs need to be differentiated from extrarenal malignant rhabdoid tumor. Unlike DEACs, rhabdoid tumors are not associated with a well-differentiated endometrioid carcinoma.

## Conclusion

In summary, DEAC is a rare and recently defined entity, which has distinct histological and immunohistochemical features and should not be underdiagnosed as conventional endometrioid adenocarcinoma. Furthermore, among the endometrioid type 2 tumors, UC including DEAC should be further categorized because of their worse clinical and biological behavior.

## Abbreviations

BMI: Body mass index
BSO: Bilateral salpingo-oophorectomy
CET1WI: Contrast-enhanced T1-weighted images
CK AE1/AE3: Pancytokeratin
CK8/18: Cytokeratin 8/18
DEAC: Dedifferentiated endometrioid adenocarcinoma
EBRT: External-beam pelvic RT
EMA: Epithelial membrane antigen
ER: Estrogen receptor
FIGO: Federation of Obstetrics and Gynecology
HPF: High-power field
ICR: Intracavitary radiotherapy
LN: Lymph node
PR: Progesterone receptor
TH: Total hysterectomy
UC: Undifferentiated carcinoma
